# A Native Nepenthesin Reactor for Improved Proteolytic Digestion of Intrinsically Disordered Proteins in Proteomics Workflows

**DOI:** 10.1002/cbic.202500832

**Published:** 2026-03-26

**Authors:** Christian Wall, Frank Hause, Wiebke Grimm, Florian W. Otto, Erik Siefke, Marc Kipping, Andrea Sinz

**Affiliations:** ^1^ Department of Pharmaceutical Chemistry and Bioanalytics Martin Luther University Halle‐Wittenberg Halle Germany; ^2^ Center for Structural Mass Spectrometry Martin Luther University Halle‐Wittenberg Halle Germany; ^3^ Faculty of Medicine Institute of Molecular Medicine Martin Luther University Halle‐Wittenberg Halle Germany

**Keywords:** intrinsically disordered proteins, mass spectrometry, nepenthesin, protease immobilization, proteomics

## Abstract

Intrinsically disordered proteins and proteins containing intrinsically disordered regions often harbor sequences that are difficult to digest with conventional proteases, such as trypsin, Asp‐N, or pepsin. In particular, proline‐rich regions (PRRs) resist efficient proteolysis and limit sequence coverage in proteomic workflows. Nepenthesins originate from pitcher plants, combining high catalytic activity and stability under acidic conditions with a broad substrate specificity. We describe a workflow for the extraction and purification of native nepenthesin (NEP‐NAT) from greenhouse‐cultivated *Nepenthes* species, followed by the enzyme's covalent immobilization on POROS‐AL chromatographic material. The performance of the NEP‐NAT reactor was evaluated in an online digestion liquid chromatography/tandem mass spectrometry setup for accelerated proteolysis, showing a high proteolytic activity for myoglobin, *α*‐synuclein, and insulin‐like growth factor 2 mRNA‐binding protein 1. While commercial nepenthesin columns yielded broad coverage for structured proteins, the NEP‐NAT reactor generated the largest number of peptides for the intrinsically disordered protein *α*‐synuclein. Cleavages at Pro residues showed enhanced digestion in the PRR of the tumor suppressor protein p53, where conventional proteases show limited activity. These results confirm NEP‐NAT as a potent protease in proteomics workflows, offering enhanced access to Pro‐rich and disordered domains that are largely inaccessible to common proteases.

## Graphical abstract

A workflow is presented for the extraction and purification of native nepenthesin (NEP‐NAT) from *Nepenthes* species, followed by the enzyme's immobilization on POROS‐AL chromatographic material. The NEP‐NAT reactor efficiently digested intrinsically disordered proteins and cleaved at Pro residues, outperforming commercial columns in an online LC/MS/MS setup.

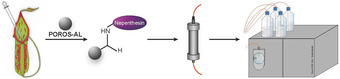

## 1 Introduction

Intrinsically disordered proteins (IDPs) and intrinsically disordered regions (IDRs) challenge the classical structure–function paradigm of protein biology [1, 2]. IDPs and IDRs play essential roles in cellular signaling, regulation, and stress adaptation, but lack a stable tertiary structure [3]. Their functional versatility arises from conformational flexibility and the capacity to engage in multiple interactions with structurally distinct partners [4, 5]. These features allow them to participate in diverse biological processes, including transcriptional regulation [6], DNA damage response and repair [7], and cellular stress response pathways [8].

From an analytical perspective, the inherent structural plasticity and amino acid composition of IDPs/IDRs pose substantial challenges for their characterization in proteomics studies. They are often enriched in polar and charged residues and depleted in hydrophobic amino acid residues [9]. In particular, proline‐rich regions (PRRs), low‐complexity sequences, and regions devoid of secondary structure elements frequently resist a proteolytic cleavage, resulting in incomplete sequence coverage and biased peptide maps [10]. Therefore, there is a pressing need for proteolytic tools with complementary cleavage preferences that are capable of efficiently processing the flexible, often Pro‐rich segments in IDPs/IDRs.

Trypsin, a serine protease widely used in bottom‐up proteomics workflows, cleaves C‐terminally to Lys and Arg residues, generating peptides with basic C‐termini that ionize efficiently and are highly compatible with tandem mass spectrometry (MS) [11]. Usually, trypsin will not cleave if a Pro residue follows Lys or Arg [12]. IDPs/IDRs, being enriched in polar, charged, and low‐complexity sequences [9], often have either sparse or very high distributions of Lys and Arg residues in the desired spacing for ideal tryptic peptides. As a result, IDPs/IDRs yield either very long peptides, peptides with few charges, or alternatively very short peptides or no cleavage at all, reducing sequence coverage. The usual enzyme for proteomics experiments is trypsin, which requires near‐neutral to slightly basic pH and moderate temperatures [13] for an efficient digestion of proteins. These conditions might lead to aggregation of IDPs/IDRs as labile protein systems. Using a variety of proteases will aid in efficiently digesting IDPs/IDRs, and the optimum set of proteases has to be tested for each protein system under investigation. IDPs/IDRs remain underrepresented in standard tryptic proteomics workflows [10] including also methods of structural proteomics, such as hydrogen/deuterium exchange mass spectrometry (HDX–MS) [14, 15], footprinting methods [16], and crosslinking MS (XL–MS) [17].

Nepenthesin,^1^ an aspartic protease, provides a powerful alternative to conventional proteases by increasing the accessibility of IDPs/IDRs for proteomics workflows. Naturally secreted by carnivorous pitcher plants of the genus *Nepenthes*, nepenthesin is a key component of the acidic digestive fluid that decomposes captured insects, enabling nitrogen acquisition in nutrient‐poor environments [18]. Nepenthesin displays remarkable biochemical stability over a wide pH range (pH 3–10) and at elevated temperatures (up to 50 °C), while maintaining high proteolytic efficiency under denaturing conditions [19]. Most importantly, it exhibits a unique and broad substrate specificity, efficiently cleaving C‐terminally at different amino acid residues, including Pro [20]. This Pro‐cleaving ability is of particular value for analyzing IDPs/IDRs with PRRs that remain inaccessible to standard proteases in proteomics workflows.

Despite these promising properties, the practical use of nepenthesin in proteomics workflows has been hampered by difficulties in obtaining active enzyme at sufficient amounts [21]. Natural concentrations in pitcher plant fluid are low and variable, while recombinant expression systems have yielded enzymes of reduced stability and compromised specificity, often lacking the crucial Pro‐directed cleavage activity [21]. This observation suggests that cofactors or posttranslational modifications unique to the plant‐derived form are essential for its optimal catalytic behavior [21].

In this study, we report a reliable strategy for the extraction, purification, and immobilization of native nepenthesin (NEP‐NAT) from greenhouse‐cultivated *Nepenthes* species on POROS‐AL chromatographic material [22]. The resulting NEP‐NAT reactor exhibits exceptional stability and activity under acidic conditions and enables highly efficient proteolysis of structurally diverse proteins, including those with IDRs. Comparative liquid chromatography/tandem mass spectrometric (LC/MS/MS) analyses of myoglobin, *α*‐synuclein, and insulin‐like growth factor 2 mRNA‐binding protein 1 (IGF2BP1) demonstrate that the native enzyme generates superior sequence coverage than commercially available nepenthesin and/or pepsin preparations. Notably, only pitcher plant‐derived nepenthesin achieved reliable proteolytic cleavage C‐terminally to Pro residues, improving digestion efficiency especially for the disordered domains of *α*‐synuclein and p53. These findings confirm NEP‐NAT as a powerful proteolytic tool for the comprehensive characterization of IDPs/IDRs, providing access to regions previously refractory to analysis and expanding the proteomics toolbox for studying the structural plasticity of the proteome.

## 2 Results and Discussion

### 2.1 Production and Immobilization of Native Nepenthesin Preparations

To identify the most suitable source for enzyme preparation, raw pitcher fluids were collected from different *Nepenthes* species (*N. alata*, *N. maxima*, and *N. truncata*). Each collection yielded 60–120 mL of raw fluid, corresponding to an average recovery of ≈30 µg purified nepenthesin per 100 mL. Raw fluids were concentrated to yield nepenthesin solutions of 10–50 µg/mL. The proteolytic activities of nepenthesin solutions were determined by quantifying the amount of proteolytic peptides generated from bovine serum albumin (BSA) over a 60 min incubation period. For details, see Fig. S1. SDS‐PAGE analysis (Fig. S2) was used to determine the purity of nepenthesin preparations. It is clearly visible from the gel that several proteins are contained in NEP‐NAT from pitcher fluid. Apparently, the proteins contained in the preparations are glycosylated as is visible after PNGase F treatment.

Across all harvests, fluids from *N. truncata* plants exhibited the highest activity, followed by those from *N. alata* and *N. maxima* plants. These results are consistent with previous reports describing a high variability of pitcher fluids among *Nepenthes* species and developmental stages, reflecting differences in enzyme composition and secretion dynamics [23, 24].

The purified fractions were concentrated by centrifugation to a final volume of 3–4 mL. In total, about 600 µg of nepenthesin was obtained, of which roughly 300 µg was used for final immobilization on aldehyde‐functionalized POROS‐AL chromatographic material (Scheme S1) [25]. POROS‐AL is widely used for protease immobilization and has been successfully applied to enzymes, such as trypsin [26], pepsin [27], and recombinant nepenthesin [22], underlining its versatility for preparing stable enzyme reactors. Immobilization was achieved by covalent coupling of nepenthesin amine groups to the POROS‐AL beads, with the resulting imines being stabilized through reductive amination using sodium cyanoborohydride ( Scheme S1). This procedure ensured stable covalent attachment of the enzyme to the matrix, and the activity of immobilized nepenthesin was verified prior to digestion of the three model proteins.

### 2.2 Comparison of Immobilized Native Nepenthesin With Commercial Enzyme Preparations

**FIGURE 1:**
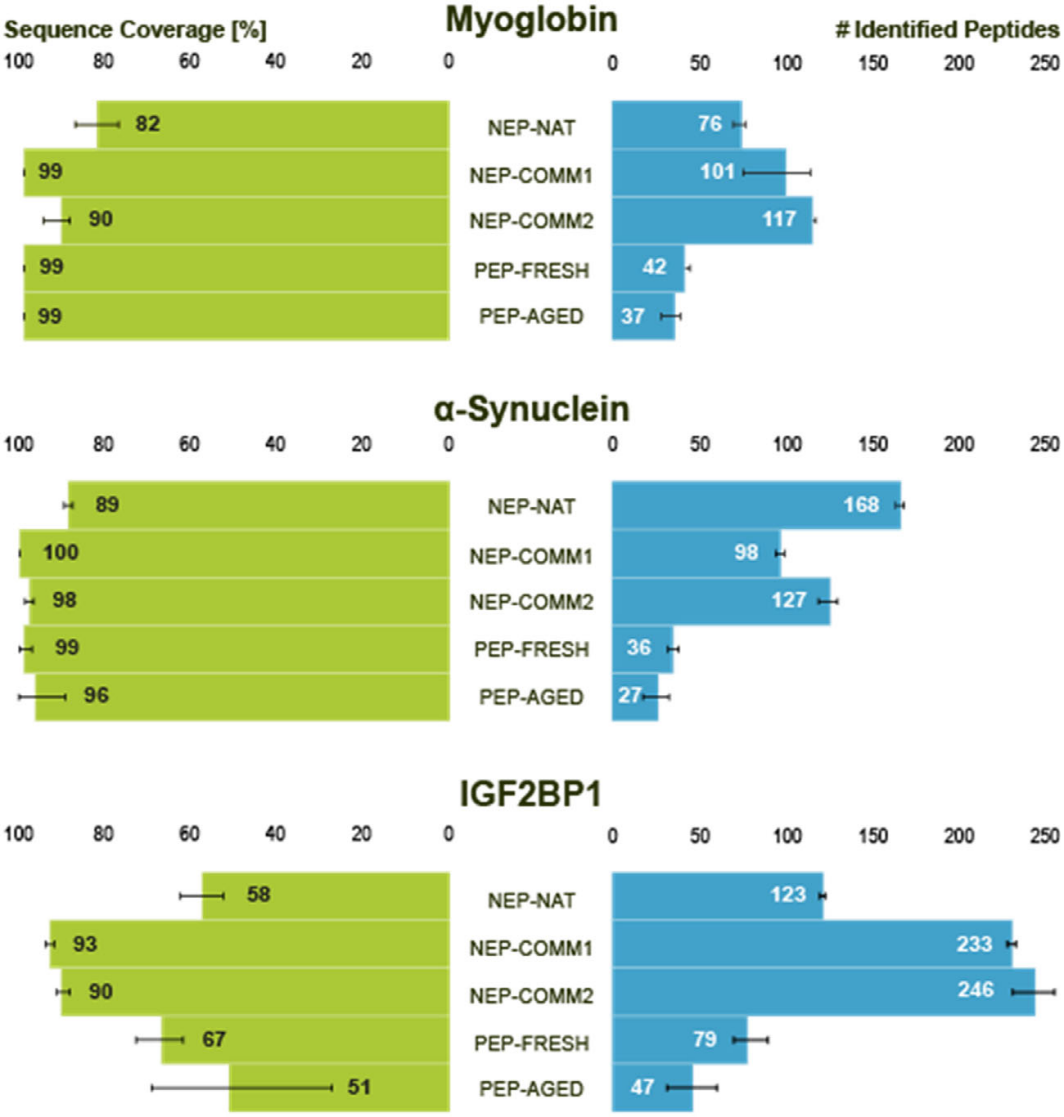
Benchmarking of different enzyme reactors. Bar plots show sequence coverage (left, green) and number of identified peptides (right, blue) for myoglobin, *α*‐synuclein, and IGF2BP1 digested with different enzyme reactors under identical LC/MS/MS conditions. Columns comprise the NEP‐NAT reactor, two commercial nepenthesin columns (NEP‐COMM1 and NEP‐COMM2), and two pepsin columns (PEP‐FRESH and PEP‐AGED). Error bars represent standard deviation from three technical replicates. Peptides were identified with PEAKS Studio 10 applying an FDR of 1%. All peptide identifications were manually validated. Error bars represent standard deviation of values from three technical replicates. LC/MS/MS = Liquid chromatography/tandem mass spectrometry; NEP‐NAT = native nepenthesin.

To evaluate the performance of the NEP‐NAT preparation in proteomics workflows, the custom‐prepared reactor containing NEP‐NAT was benchmarked against two commercial nepenthesin preparations (NEP‐COMM1, containing recombinant nepenthesin alone, and NEP‐COMM2, a mixture of nepenthesin and pepsin) as well as new and aged pepsin columns (PEP‐FRESH and PEP‐AGED). The NEP‐NAT reactor was integrated in an online digestion LC/MS/MS setup for accelerated proteolysis (Supporting Information (SI)). The performance of the reactor was compared to pepsin columns as both enzymes cleave proteins unspecifically. Three proteins were selected for digestion, myoglobin, *α*‐synuclein, and insulin‐like growth factor 2 mRNA binding protein 1 (IGF2BP1) (for amino acid sequences, see SI), to compare proteolytic efficiency under identical LC/MS conditions. The number of peptides generated and overall sequence coverage served as indicators for digestion performance (Figure 1).

Across the three proteins, all nepenthesin columns (NEP‐NAT, NEP‐COMM1, and NEP‐COMM2) consistently produced a higher number of peptides compared to pepsin columns (PEP‐FRESH and PEP‐AGED), highlighting the superior cleavage efficiency of nepenthesin. For IGF2BP1, NEP‐COMM1 and NEP‐COMM2 yielded the largest number of peptides and highest sequence coverage. In contrast, for the IDP *α*‐synuclein, the NEP‐NAT reactor outperformed all other columns, generating the highest number of peptides. This indicates a particular advantage of NEP‐NAT in processing highly flexible, disordered regions that are often inaccessible to conventional proteases.

In terms of overall sequence coverage, the NEP‐NAT reactor did not fully reach the levels obtained with NEP‐COMM1 and NEP‐COMM2, suggesting that NEP‐NAT preparations might be beneficial as complementary proteases in addition to established enzymes.

Regarding the number of identified proteolytic peptides, NEP‐COMM1 and NEP‐COMM2 columns performed best for myoglobin and IGF2BP1, while the NEP‐NAT reactor produced the largest number of peptides for the IDP *α*‐synuclein.

### 2.3 Application of Nepenthesin for Proteolytic Digestion of Intrinsically Disordered Proteins/Intrinsically Disordered Regions

**FIGURE 2:**
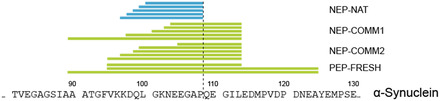
Proteolytic characteristics of a NEP‐NAT reactor. Peptides identified from *α*‐synuclein digestion show that exclusively the NEP‐NAT reactor cleaved C‐terminally at a specific Pro residue (dotted line).

To assess whether pitcher plant‐derived nepenthesin offers distinct advantages for IDPs/IDRs, the digestion of *α*‐synuclein was examined, which contains five proline residues in its C‐terminal IDR (SI). A closer inspection of the proteolytic peptides revealed that at the exemplary displayed position 108, exclusively the NEP‐NAT column was capable of cleaving C‐terminally at a specific Pro residue (Figure 2). A closer inspection of the proteolytic peptides revealed that exclusively the NEP‐NAT column was capable of cleaving C‐terminally at a specific Pro residue (Figure 2). The proteolytic peptides generated with commercial pepsin preparations were larger as pepsin usually disfavors cleavage C‐terminally at Pro residues [12], which limits digestion efficiency for many IDPs/IDRs. In contrast, NEP‐NAT has been described to support cleavage after residues that are considered “forbidden” for pepsin (K, R, H, and P) [28].

The absence of cleavage at that specific Pro residue with the commercial NEP‐COMM1 and NEP‐COMM2 columns is consistent with reports that recombinant nepenthesin preparations retain their proteolytic activity at low pH [20]. However, they fail to generate C‐terminal Pro cleavages as observed for NEP‐NAT preparations. This points to an origin‐dependent specificity, where native enzyme preparations may differ from recombinant ones in isoform composition, posttranslational modifications, or minor amounts of copurified proteases.

A further plausible contributor to facilitate C‐terminal cleavage at Pro residues in pitcher plant‐derived *Nepenthes* fluids is neprosin, a Pro‐endoprotease known to cleave efficiently after Pro residues even at low pH [29, 30]. Its presence in native pitcher plant fluids provides a mechanistic explanation for the presence of peptides with C‐terminal Pro residues in NEP‐NAT digests. Whether the observed cleavages arise directly from nepenthesin or from coextracted neprosin activity cannot be resolved from the present data. Nonetheless, NEP‐NAT preparations clearly enable access to Pro‐rich IDRs that remain largely inaccessible to pepsin and the commercial nepenthesin reactors tested herein.

**FIGURE 3:**
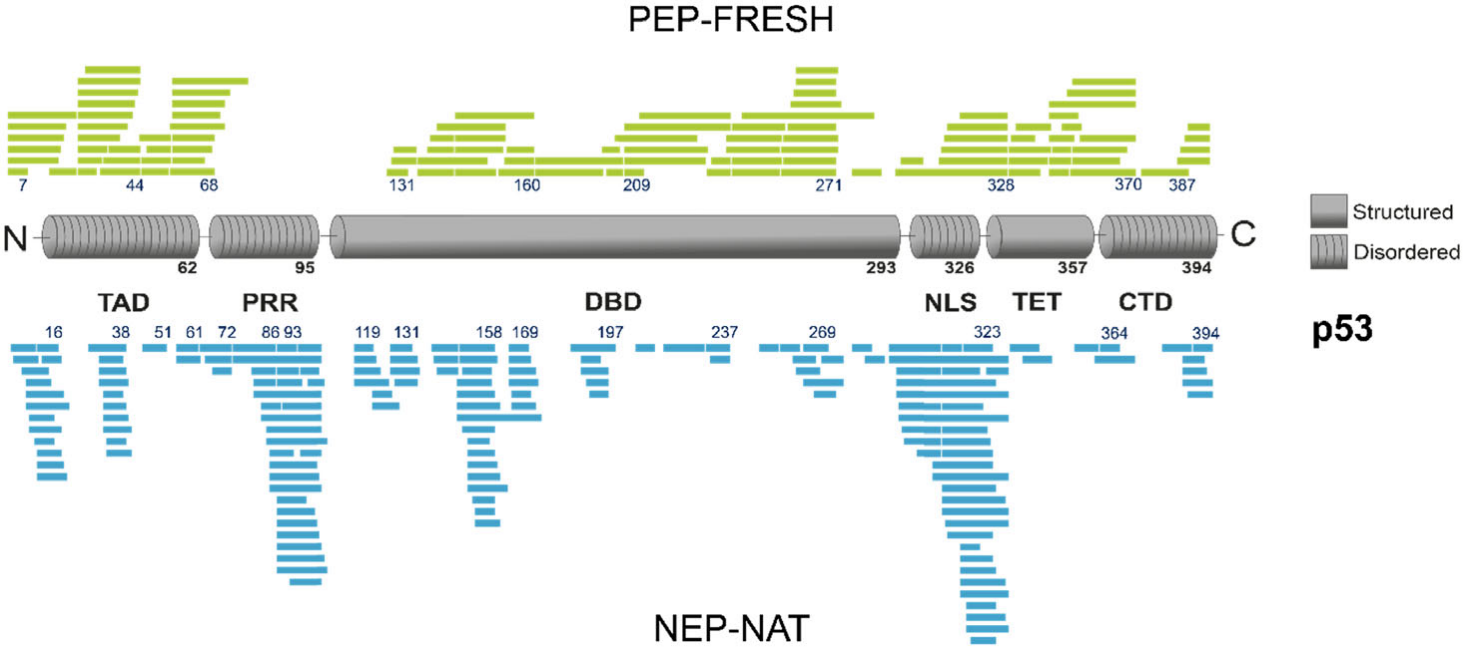
Proteolytic cleavage patterns of nepenthesin and pepsin for the tumor suppressor p53. Schematic representation of peptides generated from p53 digestion using the PEP‐FRESH column (green, top) or the native nepenthesin reactor NEP‐NAT (blue, bottom). Only the NEP‐NAT reactor showed cleavage at residues Pro‐61, Pro‐72, Pro‐86, and Pro‐93 of p53. NEP‐NAT = Native nepenthesin.

To evaluate the applicability of NEP‐NAT preparations for IDPs/IDRs with respect to (structural) proteomics workflows, digestion experiments were extended to p53. The tumor suppressor p53 is one of the most prominent IDPs with circa 40% of its sequence being disordered [31]. A distinct feature of p53 is the PRR in its N‐terminal region. Additional IDRs include the transactivation domain and the nuclear localization signal‐containing domain (NLS). Similar to *α*‐synuclein, the NEP‐NAT reactor generated a broad range of peptides originating from cleavages within the PRR and the NLS, while the PEP‐FRESH column produced almost no peptides from the PRR (Figure 3).

In contrast, pepsin digestion performed better in structured parts of the protein, particularly in generating peptides from the C‐terminal region of the DNA‐binding domain, the tetramerization domain, and the C‐terminal domain. These results emphasize the complementary proteolytic characteristics of NEP‐NAT preparations and pepsin. While pepsin remains highly effective for compact, structurally well‐ordered domains, NEP‐NAT apparently provides unique access to Pro‐rich IDRs that are otherwise largely resistant to proteolytic cleavage.

## 3 Conclusion

We present an integrated workflow for the extraction, purification, and immobilization of NEP‐NAT from greenhouse‐cultivated *Nepenthes* species. The NEP‐NAT reactor was used in an online digestion LC/MS/MS setup for accelerated proteolysis, enabling its straightforward integration in standard proteomics workflows. NEP‐NAT showed high digestion efficiency under acidic conditions, as they are required in HDX–MS experiments, consistently outperforming pepsin in peptide yield and providing unique access to PRRs in IDPs/IDRs that are otherwise poorly accessible with conventional proteases. Benchmarking against commercial nepenthesin reactors revealed clear origin‐dependent differences. While recombinant enzyme preparations yielded broad coverage for globular proteins, the NEP‐NAT reactor enabled cleavage at Pro residues in *α*‐synuclein and in the PRR of p53.

These findings confirm NEP‐NAT as a valuable addition to the proteomics toolbox. Its complementary specificity to pepsin broadens peptide diversity, enhances analytical access, and improves the characterization of IDPs/IDRs. The combined use of different proteases together with NEP‐NAT provides a powerful strategy to assess optimal sequence coverage to study structurally heterogeneous proteins.

## Supporting Information

Additional Supporting Information can be found online in the Supporting Information section. **Supporting Scheme S1**: Reaction scheme of immobilizing nepenthesin on aldehyde‐activated POROS‐AL resin. Primary amine groups, such as in lysine residues, react with aldehyde groups of the POROS‐AL resin forming imines that are then reduced to secondary amines by sodium cyanoborohydride. **Supporting** **Fig. S1**: The activity of nepenthesin was determined based on the proteolytic BSA peptides based on their UV absorption at 280  nm. BSA (2  mg/ml) was digested with nepenthesin (34 µg/ml) and samples were taken at different time points. **Supporting** **Fig. S2**: SDS‐PAGE of NEP‐NAT.nes are labeled as (M): molecular weight marker, numbers indicate molecular weight in kDa; (1): 5 µg nepenthesin; (2): 5 µg nepenthesin treated with PNGase F (2 µl, Promega, cat. no. V483A). Bands I–VII were excised from the gel, digested with chymotrypsin at 37°C for 4  h and analyzed by nano‐HPLC/nano‐ESI–MS/MS. **Supporting** **Table S1**: Proteolytic columns used for online digestion experiments.

## Funding

This study was supported by Deutsche Forschungsgemeinschaft (RTG2467, CRC 1664), Region of Saxony‐Anhalt, Martin Luther University Halle‐Wittenberg (Center for Structural Mass Spectrometry). FWO was funded by the DFG (RTG 2751 “InCuPanC”, project number 449501615).

## Conflict of Interest

The authors declare no conflicts of interest.

## Supporting information

Supplementary Material

## Data Availability

The data that support the findings of this study are available from the corresponding author upon reasonable request.

## Endnotes

1
Nepenthesin is referred herein as the mixture of proteases isolated from pitcher plant fluid (Fig. S2).
