# Supplementary material for: A Native Nepenthesin Reactor for Improved Proteolytic Digestion of Intrinsically Disordered Proteins in Proteomics Workflows
Source: Chembiochem. 2026 Mar 26;27(6):e202500832. doi: 10.1002/cbic.202500832 (PMC13022474; doi:10.1002/cbic.202500832)
Supplement: Supplementary file 1 — Supplementary Material [file CBIC-27-e202500832-s001.pdf]

Supporting Information

A Native Nepenthesin Reactor for Improved Proteolytic Digestion of Intrinsically Disordered Proteins in Proteomics Workflows

Christian Wall[a,b] (0009-0005-6666-1738), Frank Hause[a,b,c] (0000-0002-6879-6944), Wiebke Grimm[a,b] (0009-0004-0317-5591), Florian W. Otto[a,b], Erik Siefke[a,b], Marc Kipping[a,b] (0000-0002-3763-0300), Andrea Sinz*[a,b] (0000-0003-1521-4899)

[a] Christian Wall, Frank Hause, Wiebke Grimm, Florian W. Otto, Erik Siefke, Marc Kipping, Andrea Sinz
Department of Pharmaceutical Chemistry and Bioanalytics
Martin Luther University Halle-Wittenberg
Kurt-Mothes-Str. 3
06120 Halle (Saale)
Germany
E-mail: andrea.sinz@pharmazie.uni-halle.de

[b] Christian Wall, Frank Hause, Wiebke Grimm, Florian W. Otto, Erik Siefke, Marc Kipping, Andrea Sinz
Center for Structural Mass Spectrometry
Martin Luther University Halle-Wittenberg
Kurt-Mothes-Str. 3
06120 Halle (Saale)
Germany

[c] Frank Hause
Institute of Molecular Medicine, Section for Molecular Cell Biology
Faculty of Medicine
Martin Luther University Halle-Wittenberg
Kurt-Mothes-Straße 3a
06120 Halle (Saale)
Germany

Supplementary Methods

Determination of Proteolytic Activity

The proteolytic activity of nepenthesin was determined by measuring the UV absorption at 280 nm of proteolytic peptides from bovine serum albumin (BSA, Sigma). 2 µl of concentrated nepenthesin (10-50 µg/ml) in 100 mM glycine HCl, pH 2.5 were added to 40 µl of BSA (2 mg/ml) in 100 mM glycine HCl, pH 2.5. After 5, 10, 15, 20, 30, 45 and 60 minutes, 5 µl of the BSA/nepenthesin mixture was mixed with 5 µl of 20% trichloroacetic acid (TCA, Sigma). A control sample was prepared without adding nepenthesin. Samples were kept at 4°C for 10 minutes and centrifuged at 14,000 x g for 5 minutes before determining the UV absorption at 280 nm (Nanodrop 2000 spectrophotometer, Thermo Scientific) of the proteolytic peptides in the supernatant (Fig. S1).

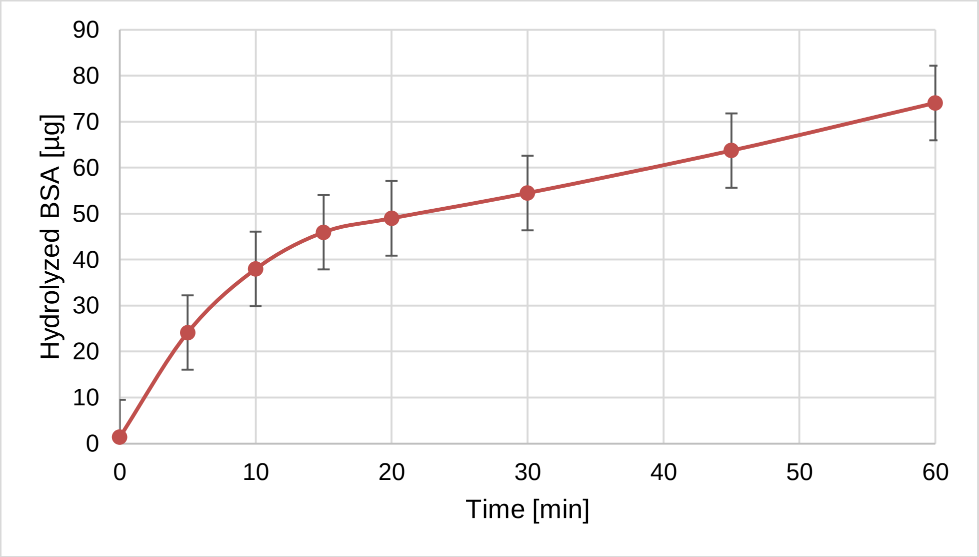

Figure S1. The activity of nepenthesin was determined based on the proteolytic BSA peptides based on their UV absorption at 280 nm. BSA (2 mg/ml) was digested with nepenthesin (34 µg/ml) and samples were taken at different time points.

SDS-PAGE Analysis of Nepenthesin Preparations

SDS-PAGE analysis (Fig. S2) was used to determine the purity of the nepenthesin preparations. It is clearly visible from the gel that several proteins are contained in native nepenthesin mixtures. Apparently, the proteins in the preparations are glycosylated as is visible by the shift of bands to lower apparent molecular weights after PNGase F treatment. Identification of bands I-VII by nano-HPLC/nano-ESI-MS/MS (Orbitrap Q-Exactive Plus) yielded the following proteins in varying amounts: Nepenthesin-1, nepenthesin-2, nepenthesin-3, two C-terminal peptidases, aspartic protease, two chitinases, and RNAse T2.

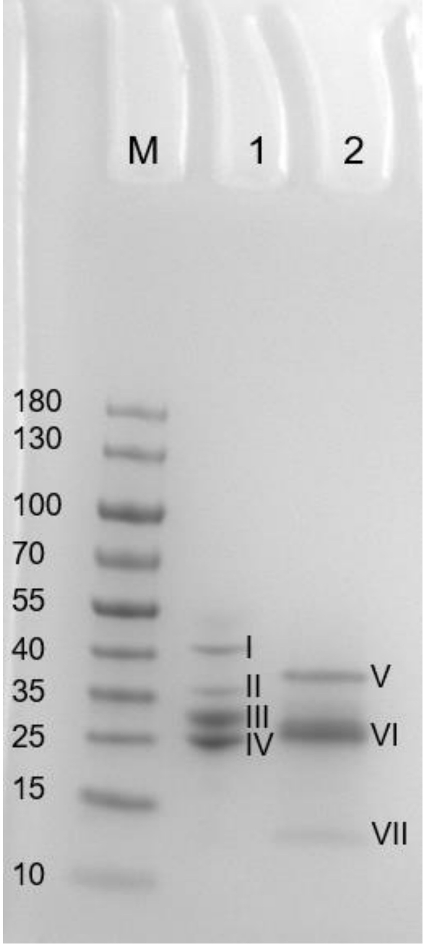

Figure S2. SDS-PAGE of native nepenthesin. Lanes are labeled as (M): molecular weight marker, numbers indicate molecular weight in kDa; (1): 5 µg nepenthesin; (2): 5 µg nepenthesin treated with PNGase F (2 µl, Promega, cat. no. V483A). Bands I-VII were excised from the gel, digested with chymotrypsin at 37°C for 4 hours and analyzed by nano-HPLC/nano-ESI-MS/MS.

Immobilization of Nepenthesin on POROS-AL Resin

Prior to immobilization, the nepenthesin solution (10-50 µg/ml) was dialyzed into 50 mM citrate buffer, pH 4. Several nepenthesin batches were pooled to give a total amount of 300 µg nepenthesin that was immobilized on aldehyde-activated POROS-AL resin (Thermo Scientific, cat. no. 1602906). The ε-amino groups of lysine residues react with the aldehyde groups of the resin to form imines that are reduced with sodium cyanoborohydride ($NaBH_3CN$) to form stable secondary amines (Scheme S1). After the coupling reaction, 50 µl of Tris-HCl buffer were added to quench remaining aldehyde groups. The resin (2 ml) is washed with 0.1% formic acid and stored in 1 ml of glycine-HCl buffer before use.

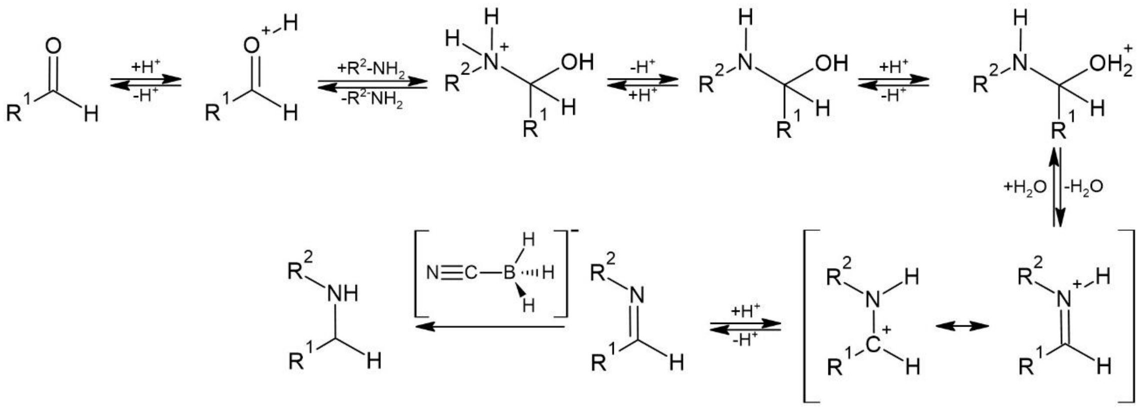

Scheme S1. Reaction scheme of immobilizing nepenthesin on aldehyde-activated POROS-AL resin. Primary amine groups, such as in lysine residues, react with aldehyde groups of the POROS-AL resin forming imines that are then reduced to secondary amines by sodium cyanoborohydride.

Proteins Used in this Study

Myoglobin from horse heart was obtained from Sigma-Aldrich (cat. no. M1882), α-synuclein,[1] IGF2BP1,[2] and p53[3] were recombinantly expressed in *E. coli* following established protocols.

The amino acid sequences of the proteins used in this study are given below.

p53 (L344A variant)

```
SMEEPQSDPS VEPPLSQETF SDLWKLLPEN NVLSPLPSQA MDDLMLSPDD IEQWFTEDPG
PDEAPRMPEA APRVAPAPAA PTPAAPAPAP SWPLSSSVPS QKTYQGSYGF RLGFLHSGTA
KSVTCTYSPA LNKMFCQLAK TCPVQLWVDS TPPPGTRVRA MAIYKQSQHM TEVVRRCPHH
ERCSDSDGLA PPQHLIRVEG NLRVEYLDDR NTFRHSVVVP YEPPEVGSDC TTIHYNYMCN
SSCMGGMNRR PILTIITLED SSGNLLGRNS FEVRVCACPG RDRRTEEENL RKKGEPHHEL
PPGSTKRALP NNTSSSPQPK KKPLDGEYFT LQIRGRERFE MFREANEALE LKDAQAGKEP
GGSRAHSSHL KSKKGQSTSR HKKLMFKTEG PDSD
```

α-Synuclein

```
MDVFMKGLSK AKEGVVAAAE KTKQGVAEAA GKTKEGVLYV GSKTKEGVVH GVATVAEKTK
EQVTNVGGAV VTGVTAVAQK TVEGAGSIAA ATGFVKKDQL GKNEEGAPQE GILEDMPVDP
DNEAYEMPSE EGYQDYEPEA
```

N-terminus is acetylated.

IGF2BP1

```
GPLGSPEFMN KLYIGNLNES VTPADLEKVF AEHKISYSGQ FLVKSGYAFV DCPDEHWAMK
AIETFSGKVE LQGKRLEIEH SVPKKQRSRK IQIRNIPPQL RWEVLDSLLA QYGTVENCEQ
VNTESETAVV NVTYSNREQT RQAIMKLNGH QLENHALKVS YIPDEQIAQG PENGRRGGFG
SRGQPRQGSP VAAGAPAKQQ QVDIPLRLLV PTQYVGAIIG KEGATIRNIT KQTQSKIDVH
RKENAGAAEK AISVHSTPEG CSSACKMILE IMHKEAKDTK TADEVPLKIL AHNNFVGRLI
GKEGRNLKKV EQDTETKITI SSLQDLTLYN PERTITVKGA IENCCRAEQE IMKKVREAYE
NDVAAMSLQS HLIPGLNLAA VGLFPASSSA VPPPPSSVTG AAPYSSFMQA PEQEMVQVFI
PAQAVGAIIG KKGQHIKQLS RFASASIKIA PPETPDSKVR MVIITGPPEA QFKAQGRIYG
KLKEENFFGP KEEVKLETHI RVPASAAGRV IGKGGKTVNE LQNLTAAEVV VPRDQTPDEN
DQVIVKIIGH FYASQMAQRK IRDILAQVKQ QHQKGQSNQA QARRK
```

Myoglobin

```
MGLSDGEWQQ VLNVWGKVEA DIAGHGQEVL IRLFTGHPET LEKFDKFKHL KTEAEMKASE
DLKKHGTVVL TALGGILKKK GHHEAELKPL AQSHATKHKI PIKYLEFISD AIIHVLHSKH
PGDFGADAQG AMTKALELFR NDIAAKYKEL GFQG
```

Online Digestion Liquid Chromatography Tandem Mass Spectrometry

The individual proteolytic columns used for online digestion experiments are summarized in Table S1. Proteins were digested online using an HP 1200 HPLC system (Agilent) at 4 °C. For each analysis, 50 pmol of protein were injected and loaded onto the proteolytic column using 0.1% formic acid at a flow rate of 10 µl/min for 10 minutes. After digestion, the proteolytic peptides were immediately loaded on the trap column (X-Bridge Peptide BEH C18, 2.1 x 5 mm, 2.5 µm, 300 Å, Waters). A 6-minute gradient ranging from 3% to 50% acetonitrile (+0.1% formic acid) was applied to elute the peptides from the trap column onto the separation column (X-Brigde Peptide BEH C18, 1 x 100 mm, 3.5µm, 300 Å, Waters) at a flow rate of 80 µl/min. To elute residual peptides, the trap column was flushed with 85% acetonitrile (+0.1% formic acid), followed by re-equilibration of the column at 3% acetonitrile (+0.1% formic acid) for 2 min.

Table S1. Proteolytic columns used for online digestion experiments.

Column	Enzyme Composition	Column Type	Dimensions	Supplier
NEP-NAT	Native nepenthesin (immobilized)	POROS 20 AL, in-house packed	2.1 × 20 mm	in-house
NEP-COMM1	*Nepenthesin (POROS)*	AffiPro custom column	2.1 × 20 mm	AffiPro
NEP-COMM2	*Nepenthesin/Pepsin (POROS)*	AffiPro custom column	2.1 × 20 mm	AffiPro
PEP-FRESH	Pepsin (Enzymate BEH Pepsin)	Waters Enzymate BEH Pepsin	2.1 × 30 mm	Waters
PEP-AGED	Pepsin (aged, 1 year)	Waters Enzymate BEH Pepsin	2.1 × 30 mm	Waters

The HPLC system was directly coupled to an Orbitrap Fusion Tribrid mass spectrometer equipped with ESI source (Thermo Fisher Scientific). Full MS scans were acquired in the orbitrap analyzer at R = 120,000 (*m/z* range 350–1700).

Fragmentation was carried out using higher-energy collision-induced dissociation (HCD), collision-induced dissociation (CID), electron transfer dissociation (ETD) or a combination of HCD and ETD (EThcD) with the following parameters:

- Stepped HCD-MS/MS: Priority 1; isolation window *m/z* 1.5; normalized collision energies (NCE): 27%, 30%, and 33%; orbitrap detection at R = 15,000; intensity threshold 3×10^4; charge states 1+ to 6+ were selected for fragmentation.
- CID-MS/MS: Priority 2; isolation window *m/z* 2; NCE 35%; ion trap detection in rapid scan mode at R = 15,000; intensity threshold 5×10^3; charge states 1+ to 6+ were selected for fragmentation.
- ETD/EThcD-MS/MS: Priority 1; isolation window *m/z* 1.5; reaction time 150 ms; reagent target 2.0×10^5; maximum reagent injection time 200 ms; supplemental activation with NCE 15; orbitrap detection at R = 15,000; intensity threshold 2×10^4; charge states 3+ to 6+ were selected for fragmentation.

Dynamic exclusion was enabled with the following parameters: Precursor ions were excluded for a duration of 60 s after they had been fragmented once; mass tolerance ±2 ppm.

References

[1] D. Ubbiali, M. Fratini, L. Piersimoni, C. H. Ihling, M. Kipping, I. Heilmann, C. Iacobucci, A. Sinz, “Direct Observation of "Elongated" Conformational States in α-Synuclein upon Liquid-Liquid Phase Separation” Angew Chem Int Ed Engl. **2022**, *61,* e202205726.

[2] K. Wächter, M. Köhn, N. Stöhr, S. Hüttelmaier, "Subcellular localization and RNP formation of IGF2BPs (IGF2 mRNA-binding proteins) is modulated by distinct RNA-binding domains" Biol. Chem. **2013**, *394*, 1077-1090.

[3] C. Arlt, C. H. Ihling, A. Sinz, “Structure of full-length p53 tumor suppressor probed by chemical cross-linking and mass spectrometry” Proteomics **2015**, *15*, 2746-2755.
